# Molecular characterization and subtype analysis of *Blastocystis* sp. in captive wildlife in Henan, China

**DOI:** 10.1051/parasite/2025006

**Published:** 2025-02-17

**Authors:** Zhaohui Cui, Xiyao Huang, Sulan Zhang, Kaifang Li, Aili Zhang, Qichen Li, Yutong Zhang, Junqiang Li, Meng Qi

**Affiliations:** 1 Key Laboratory of Biomarker Based Rapid-Detection Technology for Food Safety of Henan Province, Food and Pharmacy College, Xuchang University Xuchang 461000 China; 2 College of Chemical and Materials Engineering, Xuchang University Xuchang 461000 China; 3 Luohe Animal Disease Prevention and Control Center Luohe 462000 China; 4 College of Veterinary Medicine, Henan Agricultural University No. 218 of Ping An Avenue, Zhengdong Newly-developed Area Zhengzhou 450046 China; 5 College of Animal Science and Technology, Tarim University Alar 843300 China

**Keywords:** *Blastocystis* sp., Captive wildlife, Subtypes, Zoonotic potential

## Abstract

*Blastocystis* sp. is a zoonotic intestinal protozoan that is ubiquitous globally, residing in the gastrointestinal tracts of both humans and various animals. In the present study, a PCR-sequencing tool based on the SSU rRNA gene was employed to investigate the prevalence and subtypes of *Blastocystis* spp. in 204 fresh fecal samples collected from 20 captive wildlife species from a bird park in Henan Province, Central China. Overall, *Blastocystis* was present in 13.73% (28 out of 204) of the samples and 25% (5 out of 20) of the species. A total of four zoonotic subtypes of *Blastocystis* sp. were found: ST1, ST3, ST5, and ST27, with the latter being the most prevalent, accounting for 35.71% (10 out of 28) of the 5 species positive for *Blastocystis* sp. To the best of our knowledge, this is the first report of *Blastocystis* ST27 in birds in China, namely bar-headed goose (*Anser indicus)* and peafowl (*Pavo muticus*). The data suggest that captive wildlife, particularly those in bird parks, may frequently be infected with this zoonotic pathogen. Consequently, these animals may serve as potential reservoirs for zoonotic infections in humans.

## Abstract (fr)

*Blastocystis* sp. est un protozoaire intestinal zoonotique omniprésent dans le monde entier, résidant dans le tractus gastro-intestinal des humains et de divers animaux. Dans la présente étude, un outil de séquençage par PCR basé sur le gène SSU rRNA a été utilisé pour étudier la prévalence et les sous-types de *Blastocystis* spp. dans 204 échantillons de matières fécales fraîches prélevés sur 20 espèces sauvages en captivité dans un parc ornithologique de la province du Henan, en Chine centrale. Dans l’ensemble, *Blastocystis* était présent dans 13,73 % (28 sur 204) des échantillons et dans 25 % (5 sur 20) des espèces. Au total, quatre sous-types zoonotiques de *Blastocystis* sp. ont été trouvés : ST1, ST3, ST5 et ST27, ce dernier étant le plus répandu, représentant 35,71% (10 sur 28) des 5 espèces positives pour *Blastocystis* sp. À notre connaissance, il s’agit du premier signalement de *Blastocystis* ST27 chez des oiseaux en Chine, à savoir l’oie à tête barrée (*Anser indicus*) et le paon (*Pavo muticus*). Les données suggèrent que la faune en captivité, en particulier celle des parcs ornithologiques, peut être fréquemment infectée par ce pathogène zoonotique. Par conséquent, ces animaux peuvent servir de réservoirs potentiels d’infections zoonotiques pour l’homme.

## Introduction

*Blastocystis* sp. is a common single-celled gastrointestinal protozoan found in humans and many other mammalian species [5]. It is estimated that over one billion individuals worldwide are colonized or infected with *Blastocystis* sp. [6]. The potential positive effect of this organism on the gut microbiome, as well as the situations in which its presence causes symptomatic infection, is being intensively studied [9, 25]. The transmission of *Blastocystis* primarily occurs *via* the fecal-oral route or through indirect contact, resulting in a higher prevalence rate in places with poor sanitary conditions [1]. Moreover, certain subtypes of *Blastocystis* sp. exhibit a high prevalence among individuals with irritable bowel syndrome (IBS), resulting in a variety of gastrointestinal symptoms, including diarrhea, nausea, and abdominal pain [7, 28]. In general, the manifestation and severity of clinical symptoms, as well as the disease’s overall impact, are influenced by various subtypes of *Blastocystis* [2, 10, 23].

*Blastocystis* is well-known for its morphological polymorphism and genetic diversity, and there is a likelihood of discovering more subtypes in the future [32]. To date, the subtyping of *Blastocystis* has enabled the identification of at least 44 proposed subtypes (STs) at the SSU rDNA locus, including some of the most widely recognized and accepted subtypes, such as ST1–ST17, ST21, and ST23–ST48 [18, 31, 32]. Among these subtypes, 17 have been documented in humans, including ST1 to ST10, ST12, ST14–ST16, ST23, ST35, and ST41 [12, 26]. These subtypes have also been identified in other mammals and birds, suggesting the potential for zoonotic transmission [5, 30]. Although *Blastocystis* STs lack host specificity, ST1–ST3 are most commonly identified in both humans and NHPs (non-human primates), while ST6 and ST7 are primarily associated with avian species, especially poultry, and are generally considered avian-adapted subtypes [29, 38]. ST10 and ST14, on the other hand, are most frequently found in ungulates [15, 27]. Therefore, accurately identifying the subtypes of *Blastocystis* in different hosts through molecular characterization is crucial for understanding its zoonotic transmission and its impact on public health.

The Bird Park is a bird culture theme park located in Henan Province, covering an area of over 600 acres. It has been rated as a national 4A level tourist attraction. Visitors have the opportunity to engage directly with wild animals in captivity, which poses a higher risk of zoonotic *Blastocystis* infection. A previous study showed that the same genetic variants are present simultaneously among animals and their caretakers, as well as in non-human primates’ zookeepers, providing strong evidence for the transmission of *Blastocystis* from animals to humans and *vice versa* [17].

The objectives of this study were to investigate the occurrence and genetic diversity of *Blastocystis* in captive wild animals at the Bird Park in Henan Province, Central China, and to evaluate the potential risk of zoonotic transmission between humans and animals.

## Materials and methods

### Ethics statement

Before initiating this study, the research protocol was reviewed and approved by the Ethics Review Committee of Xuchang University. The collection of stool samples was conducted following the acquisition of appropriate authorization from the zoo managers. It is important to note that no animals were subjected to any form of harm during the collection process of fecal samples.

### Study sites and collection of animal samples

A total of 204 fresh fecal samples were collected from a variety of captive wild animals from October to December, 2023. Each fecal sample was collected from a single individual animal. Fecal samples from birds and other animals were collected using swabs for birds and sterile gloves for other animals and were then preserved in sterile centrifuge tubes of 15 mL, and labeled with the region of collection and the animal species. The fecal samples were securely packaged with ice packs and transported to the laboratory. Upon arrival, all samples were stored at −20 °C until DNA extraction.

### DNA extraction

The samples were collected from the −20 °C freezer and washed twice with double distilled water by centrifugation at 12,000× *g* for 3 min to remove impurities, and 200 mg of sediment from each sample was used for the extraction of genomic DNA, using a TIANamp Stool DNA kit (TIANGEN, Beijing, China), according to the manufacturer’s specifications. Each DNA extraction product was stored at −20 °C for subsequent PCR amplification.

### PCR amplification and subtype identification

The prevalence and genotypes of *Blastocystis* sp. were examined using PCR, specifically targeting the region of approximately 600 bp of the SSU rRNA gene [19]. The primers used in this study were: RD5: 5′–ATCTGGTTGATCCTGCCAGT–3’; BhRDr: 5′–GAGCTTTTTAACTGCAACAACG–3′. The annealing temperature was set at 55 °C. The PCR mixture (25 μL) contained 2.5 μL 10× ExTaq buffer (Mg^2+^ free), 1.5 mM MgCl_2_, 0.2 mM deoxyribonucleoside triphosphate (dNTP), 0.625 U of ExTaq DNA polymerase (Takara, Dalian, China), 2 μL genomic DNA, and 0.4 μM of each primer. Each PCR reaction included positive and negative controls. Six microliters of PCR products were subjected to electrophoresis using 1.5% agarose gel electrophoresis and visualized under UV light after staining with Exred (Zoman, Beijing, China).

### Nucleotide sequencing and phylogenetic analysis

The amplified PCR products, approximately 600 base pairs in length, were subjected to sequencing using the primer RD5/BhRDr, in the process of PCR amplification conducted by Sangon Biotech (Shanghai, China) using an ABI 730 Auto-sequencer. The obtained nucleotide sequences were subsequently edited with Clustal X (http://www.clustal.org) and Chromas Pro (http://technelysium.com.au/ChromasPro.html). To identify overlooked mixed infections and double peaks, as well as any unclear positions, the raw chromatograms of both sequences for each sample were thoroughly examined visually. The consensus sequences were then validated as *Blastocystis* SSU rRNA gene sequences through BLAST alignment NCBI (https://blast.ncbi.nlm.nih.gov/Blast.cgi) based on homology identification. Following this, the subtypes of *Blastocystis* sp. isolates were identified by an online platform: *Blastocystis* locus/sequence definitions database (https://pubmlst.org/bigsdb?db=pubmlst_blastocystis_seqdef).

Six representative sequences from this study and reference sequences download from GenBank within NCBI were aligned and analyzed with Clustal W embedded in the MEGA 7 program (http://www.megasoftware.net/). In the final dataset, there were 594 sequence sites left after trimming the alignment, and all ambiguous positions were removed for each sequence pair (pairwise deletion option). To identify *Blastocystis* sp. subtypes, the phylogenetic analysis of *Blastocystis* sp. obtained from this study was compared with the known subtypes with neighbor-joining (NJ) analyses. The Kimura 2-parameter model and bootstrap method were used to assess the robustness of the clusters using 1,000 replicates, where only branch support values above 50% were retained.

### Nucleotide sequence accession numbers

The representative nucleotide sequences of the present study were submitted to GenBank under accession numbers PQ740455–PQ740456.

## Results

### The prevalence of *Blastocystis* sp. in captive wild animals

The overall prevalence of *Blastocystis* sp. in captive wild animals was found to be 13.73% (28 out of 204). Among the 20 species examined, a significant proportion, 5 (25%), tested positive for *Blastocystis* sp., as recorded in Table 1. The species exhibiting the highest prevalence was the bar-headed goose, with a 100% (2 out of 2) positivity rate.

**Table 1:** Occurrence of *Blastocystis* sp. in captive wildlife in Henan, China.

Host	Scientific name	Prevalence and subtypes (%) (No. positive/examined)	
		*Blastocystis* sp.	Subtypes (No.)
Macaque	*Macaca mulatta*	40.00 (8/20)	ST 1(4); ST 3(4)
Crab-eating Macaque	*Macaca fascicularis*	50.00 (4/8)	ST 1(4)
Sika Deer	*Cervus nippon*	0.00 (0/8)	–
Southern Red Muntjac	*Muntiacus muntjak*	0.00 (0/4)	–
Bactrian Camel	*Camelus*	0.00 (0/8)	–
Alpaca	*Vicugna pacos*	0.00 (0/4)	–
Northern Raccoon	*Procyon lotor*	0.00 (0/2)	–
Meerkat	*Suricata suricatta*	0.00 (0/2)	
Marmot	*Marmota*	0.00 (0/4)	–
Tiger	*Panthera tigris*	0.00 (0/6)	
Ostrich	*Struthio camelus*	37.50 (6/16)	ST 5(6)
Demoiselle Crane	*Anthropoides virgo*	0.00 (0/4)	–
Siberian Crane	*Grus leucogeranus*	0.00 (0/4)	–
Pigeon	*Columba*	0.00 (0/2)	–
Black-Crowned Crane	*Balearica pavonina*	0.00 (0/2)	–
Common Crane	*Grus grus*	0.00 (0/2)	–
Turtle Dove	*Streptopelia*	0.00 (0/2)	–
Chinese hwamei	*Garrulax canorus*	0.00 (0/2)	–
Bar-Headed Goose	*Anser indicus*	100.00 (2/2)	ST 27(2)
Peafowl	*Pavo muticus*	7.84 (8/102)	ST 27(8)
Total		13.73 (28/204)	ST 1(8), ST 3(4), ST 5(6), ST 27(10)

Following closely were the crab-eating macaque, with a positivity rate of 50% (4 out of 8), and the macaque, with a 40% (8 out of 20) positivity rate. In contrast, the prevalence of *Blastocystis* sp. in the peafowl was comparatively lower, representing 7.84% (8 out of 102). It is important to note that the small sample sizes of most host species precluded the establishment of statistical significance.

### Subtype distributions of *Blastocystis* sp. in captive wild animals

Sequence alignment revealed the presence of a total of four known *Blastocystis* sp. subtypes, namely ST1, ST3, ST5, and ST27. Among these subtypes, ST27 was the most prevalent, found in 10 out of 28 positive samples (35.71%), followed by ST1 (32%, 8 out of 28), ST5 (21.42%, 6 out of 28), and ST3 (14.28%, 4 out of 28). Table 1 provides the distribution of the different *Blastocystis* sp. subtypes among the animals.

### Genetic characteristics and phylogenetic analysis of *Blastocystis* sp. subtypes

The identity analysis of the SSU rRNA gene revealed that eight sequences of ST1 isolates identified in NHPs (macaque and crab-eating macaque) were similar to those from *Macaca fascicularis philippinensis* (KY929107, from 99.65% to 100%) and *Macaca mulatta* from Bangladesh (MN3380074, from 99.83% to 100%). The four sequences of ST3 isolates identified in the macaque were similar to those from a Rex rabbit from China (OP866804, 100%). Six sequences of ST5 isolates isolated from ostriches were similar to those from sheep in China (ON062968, 100%) and an ostrich in China (MF991106, 99.66%). ST27 was isolated for the first time from the bar-headed goose in our study. The ST27 sequences revealed 100% sequence identity with MK861944 (a peafowl isolated from China, listed as a new subtype) and shares 99.32% identity with ST27 (MW887934). Similarly, the ST27 sequence isolated from the peafowl in this study also showed 100% similarity.

**Figure 1:**
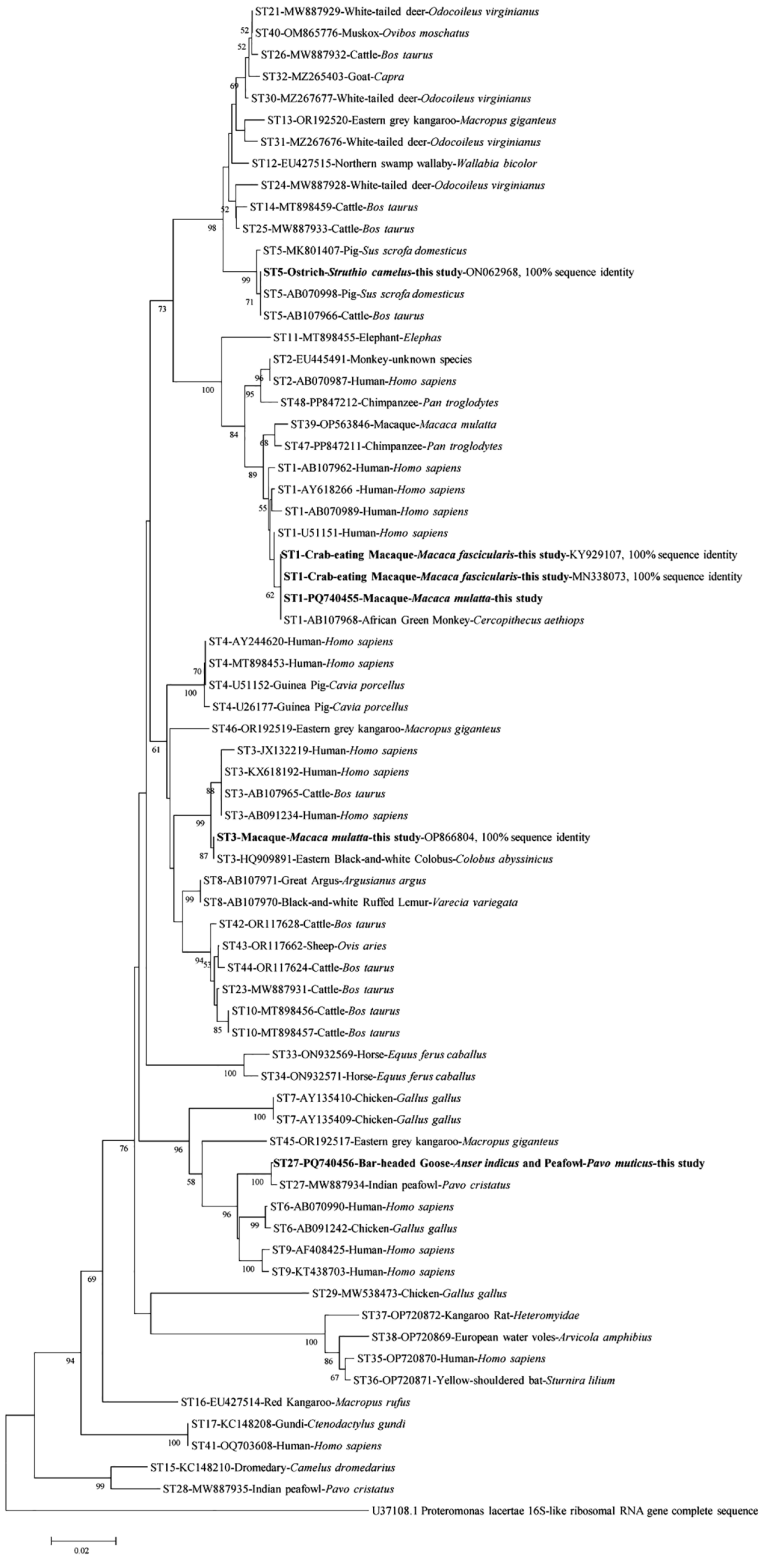
Phylogenetic relationships among nucleotide sequences of barcode regions of the small subunit ribosomal RNA (SSU rRNA) of *Blastocystis* sp.; *Blastocystis* subtypes identified in the present study are indicated in bold type.

In the current study, a total of 6 representative sequences were obtained from 28 *Blastocystis* sp. isolates. The newly acquired sequences belong to ST1, ST3, ST5, and ST27. Furthermore, the phylogenetic analysis confirmed the presence of polymorphism within the SSU rRNA gene of *Blastocystis* isolates (Fig. 1).

## Discussion

In recent decades, a multitude of researchers have engaged in comprehensive studies regarding various facets of *Blastocystis* sp., yet the debate surrounding its classification, pathogenicity, subtypes, and functions persists [4, 9, 13, 25, 30]. Consequently, a thorough comprehension of the prevalence and genetic diversity of *Blastocystis* sp. across diverse host populations globally is essential for unraveling the enigma associated with this organism. This study aimed to address this gap by analyzing the prevalence and subtype distribution of *Blastocystis* sp. among captive wildlife species at the Bird Park, Henan Province, for the first time.

In the context of this study, which encompassed a total of 20 species of captive wild animals, we found that 25% (5 out of 20) tested positive for *Blastocystis* sp. (Table 1). The overall occurrence of *Blastocystis* sp. in wild animals in captivity, specifically birds at the Bird Park, was recorded at 13.73%, which is comparable with the findings in wild animals from Colombia (13.0%) [8] and wild birds in Brazil (14.7%) [21], yet lower than in domestic and zoo animals in Algeria (38.9%) [14], animals across five zoos in China (24.5%) [3], wild primates in six European zoos (20.3%) [17], and higher than in free-ranging wild birds in Xinxiang, China (1.5%) [20], and pet birds in China (0.8%) [34]. The variations in prevalence could be attributed to sampling size, detection methods, and susceptibilities of livestock to *Blastocystis* sp. The prevalence of *Blastocystis* sp. varies among different animals, which may be cause by differences in sample collection from different hosts.

Four *Blastocystis* STs., namely ST1, ST3, ST5, and ST27, were identified in this study from 28 samples collected from captive wild animals. Among the identified subtypes, ST1 and ST3 were observed in non-human primates (NHPs), namely macaque and the crab-eating macaque. Generally, ST1-3 has frequently been determined in humans and NHPs, and represents 95% of human infections [33, 40]. In addition, a recent study has shown that zoo animals and staff have been infected with the ST1-3 subtype of *Blastocystis* sp., a finding that is in alignment with previously documented sequences from NHPs [24]. Therefore, zoos are the primary areas where zoonotic transmission of *Blastocystis* sp. from NHPs may occur.

To date, 13 subtypes (ST1, ST3-10, ST14, and ST23-25) of *Blastocystis* have been detected in birds (Greater White-Fronted Goose, White Stork, Oriental White Stork, Bean Goose, Red crowned crane, Black Swan, Ruddy Shelduck, Green peafowl, Ostrich, Canary, White-rumped munia, Budgerigar, Blue-eared pheasant, Hotan Black chickens, pigeons, and Whooper swan) in China, and ST7 is the dominant subtype among them [3, 11, 20, 36, 38]. It is worth mentioning that researchers recently reported a new discovery, in which they detected *Blastocystis* in bar-headed geese and confirmed it as ST7 through molecular identification [37]. Since the full-length SSU rRNA gene reference sequence of ST27 (MW887934) has been reported, its barcode region can now be compared with other barcode sequences available in GenBank [21, 22]. The ST27 sequences isolated from bar-headed geese revealed 100% sequence identity with the ST27 sequence isolated from peafowls within the same study, and sharing 99.32% identity with reference sequence of ST27. The phylogenetic analysis clearly demonstrated that the sequences isolated from bar-headed geese and peafowls clustered with the reference sequence of ST27 (MW887934). Distinctly, in the present study, based on our current knowledge, this is the first report of *Blastocystis* ST27 in birds (bar-headed geese and peafowls) in China.

Further, the remaining six ST5 sequences isolated from ostriches have been described previously [35, 39]. ST5 was the most predominant subtype in pigs, but it was also identified in various animals, such as NHPs, cattle, sheep, rodents, and birds [16]. Notably, the detection of all zoonotic subtypes in the current study suggests a potential transmission risk of *Blastocystis* to humans *via* contact with feces.

## Conclusions

This study documented the presence of *Blastocystis* sp. infections in various captive wildlife species within a bird park located in Henan Province, Central China. The prevalence of these infections was found to be 13.73% (28 out of 204 samples) and 25% (5 out of 20 species), marking the first identification of *Blastocystis* sp. ST27 in bar-headed geese and peafowls. Furthermore, three distinct zoonotic subtypes of *Blastocystis* sp. (ST1, ST3, and ST5) were identified across seven different host species, indicating that avian parks may serve as potential reservoirs for zoonotic *Blastocystis* sp. infections in humans. These findings provide fundamental data for a deeper understanding of the transmission dynamics of *Blastocystis* sp.
